# Image Synthesis in Nuclear Medicine Imaging with Deep Learning: A Review

**DOI:** 10.3390/s24248068

**Published:** 2024-12-18

**Authors:** Thanh Dat Le, Nchumpeni Chonpemo Shitiri, Sung-Hoon Jung, Seong-Young Kwon, Changho Lee

**Affiliations:** 1 Department of Artificial Intelligence Convergence, Chonnam National University, Gwangju 61186, Jeollanam-do, Republic of Korea; lethdat_biel@jnu.ac.kr (T.D.L.); nchum@jnu.ac.kr (N.C.S.); 2 Department of Hematology-Oncology, Chonnam National University Medical School, Chonnam National University Hwasun Hospital, Hwasun 58128, Jeollanam-do, Republic of Korea; shglory@hanmail.net; 3 Department of Nuclear Medicine, Chonnam National University Medical School, Chonnam National University Hwasun Hospital, Hwasun 58128, Jeollanam-do, Republic of Korea; kwonsy@jnu.ac.kr

**Keywords:** nuclear medicine imaging, synthesizing, transforming

## Abstract

Nuclear medicine imaging (NMI) is essential for the diagnosis and sensing of various diseases; however, challenges persist regarding image quality and accessibility during NMI-based treatment. This paper reviews the use of deep learning methods for generating synthetic nuclear medicine images, aimed at improving the interpretability and utility of nuclear medicine protocols. We discuss advanced image generation algorithms designed to recover details from low-dose scans, uncover information hidden by specific radiopharmaceutical properties, and enhance the sensing of physiological processes. By analyzing 30 of the newest publications in this field, we explain how deep learning models produce synthetic nuclear medicine images that closely resemble their real counterparts, significantly enhancing diagnostic accuracy when images are acquired at lower doses than the clinical policies’ standard. The implementation of deep learning models facilitates the combination of NMI with various imaging modalities, thereby broadening the clinical applications of nuclear medicine. In summary, our review underscores the significant potential of deep learning in NMI, indicating that synthetic image generation may be essential for addressing the existing limitations of NMI and improving patient outcomes.

## 1. Introduction

In modern healthcare, nuclear medicine imaging (NMI)—which involves using a trivial, controlled quantity of radiodiagnostic reagents—stands out as a vital tool for medical diagnosis and therapy. The reagents are designed to bind with chemical carriers that travel to certain parts of the body or accumulate through different physiological pathways linked to neoplasia. This allows for a more targeted approach to treatment [1,2]. The US Food and Drug Administration has approved a list of radiopharmaceuticals that emit three types of radiation: alpha rays, beta rays, and gamma rays [3]. Several types of radioisotope sensing are used for imaging in nuclear medicine. These include scintigraphy planar imaging, single-photon emission-computed tomography (SPECT) [4], and positron emission tomography (PET) [5]. NMI, which involves visualizing organ functions, provides detailed and functional insights for cancer detection [6,7], cardiovascular monitoring [8], bone scintigraphy [9,10], thyroid radioiodine scanning [11,12], renal scintigraphy [13], ventilation/perfusion scanning [14], liver and spleen scintigraphy [15], and neurological disorders [16,17].

The rapid progress in big data management and diagnostic program development for modern healthcare over the last few decades has required medical doctors and professional technicians to invest significant time and resources in training and processing, tasks that would be impossible without the use of increasingly complex and innovative algorithms. The growth of deep learning (DL) has attained exceptional performance and exhibited skills that exceed those of humans in numerous applications. With processes designed as a pipeline, DL algorithms can be developed to exploit all available information through techniques including abnormality detection [18,19], segmentation [20,21,22], computer-aided diagnosis [23,24], and denoising to improve image quality [25,26].

**Figure 1:**
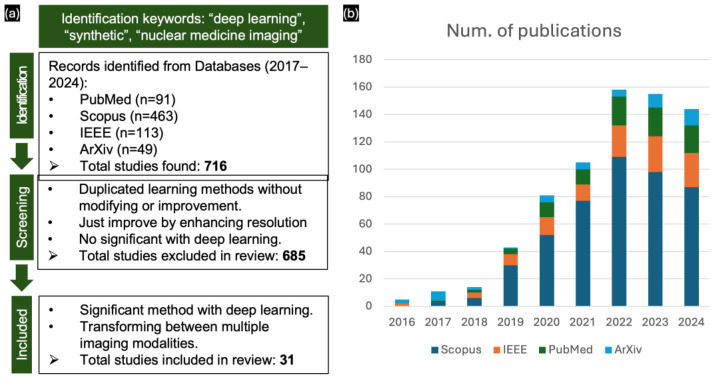
(**a**) PRISMA flow diagram of included studies. (**b**) Number of publications on NMI with deep learning from 2017 to 2024 on PubMed, Scopus, IEEE, and ArXiv.

Particularly with clinical protocols, NMI provides functional and safe doses of radiotracers, enabling effective treatment planning by radiologists and clinicians. However, using radiotracers at low doses, which is crucial for minimizing the radiation exposure to patients, often results in noisy, low-quality images, which affect diagnostic accuracy. In most scenarios, it is challenging to accurately present the complete imaging processes associated with a patient’s condition [27,28], and digital records may be lost in clinicals studies [29]. To address these challenges, DL approaches in image synthesis can be employed during acquisition and reconstruction to enhance image quality by simulating the realistic structure and presenting the specific pathologies assessing diagnostic workflows [30,31]. Thus, image synthesis utilizing DL methods has improved the capabilities and expanded the therapeutic potential of nuclear medicine, thereby cementing its position in contemporary medical practice [32,33] and facilitating advancements in multi-modal registration applications [34]. This is reflected in the publication trends from 2017 to October 2024 for the keywords “deep learning”, “synthetic”, and “nuclear medicine imaging” on PubMed, Scopus, IEEE, and ArXiv (Figure 1b). The number of publications has been increasing by approximately 20 every year, indicating rapid advancements in nuclear medicine.

This review is intended to provide an introductory outline of the combination of NMI and DL in research. We begin by reviewing the most recent improvements before reviewing the use of image translation with respect to specific research topics. Through extensive coverage, we assess the potential applications of synthetic NMI in the most recent developments.

## 2. Background

### 2.1. Scintigraphy (2D) and Tomography (3D) Through NMI

**Figure 2:**
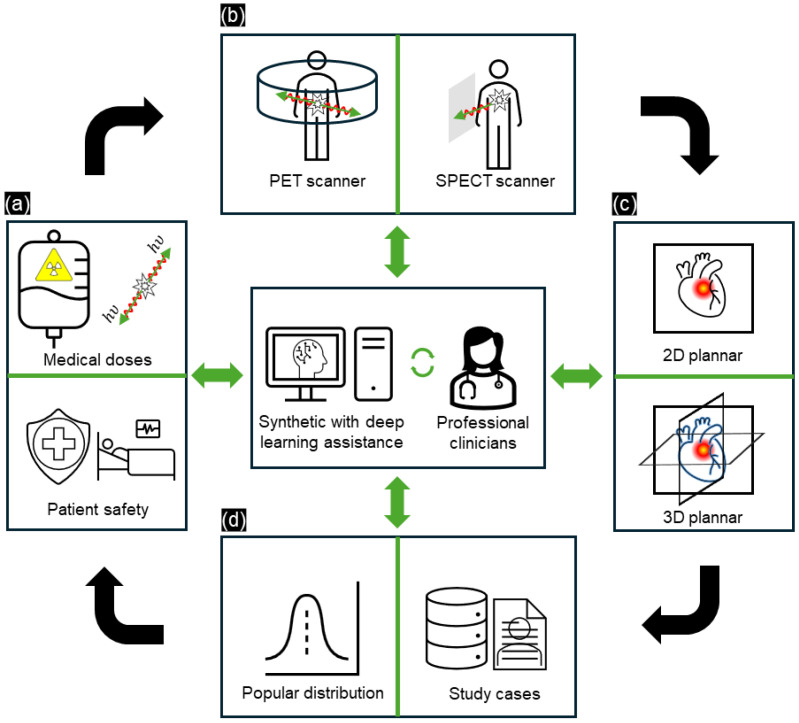
Overview of the principle of current NMI processes with DL assistance for professional clinicians. (**a**) Dose measuring and patient safety balance, (**b**) scanning geometry (scintigraphy/SPECT/PET), (**c**) reconstruction and visualization (2D/3D), and (**d**) data and study cases. The process is enhanced by incorporating DL methods, updating the knowledge of professional clinicians, and utilizing popular reports based on study cases.

The operating protocols for NMI are specified in the guidelines and regulations established by professional societies [35] (Figure 2a). The advancement of PET and SPECT systems is crucial in NMI as these systems provide insights into the complex physiological and molecular activity of radiopharmaceutical tracers. These tracers emit gamma rays, enabling PET and SPECT to visualize molecular activities (Figure 2b). In SPECT, the gamma rays emitted by the tracers are captured by gamma cameras, which rotate to acquire multiple 2D images from different angles; these images are then reconstructed into 3D datasets. In comparison, PET detects gamma rays released due to positron annihilation, offering more detailed and precise 3D visualization of molecular activity (Figure 2c). Another consideration is that nuclear medicine depends on various patient-specific factors (age, sex, family-related, etc.), with per-case differences necessitating personalized treatment (Figure 2d). For example, Xia et al. [30] provided a proposed I2-GAN network to recovery missing cardiac MRI imputation by training it with 4848 subjects within a single cardiac phase from several population-based studies. Xia’s research presented the relation between population cardiac MRI slides and provided the missing data by using a synthesis image generation model with a high SSIM value (0.872) when evaluating 100 publicly available ACDC datasets, and also helped to segment the left/right myocardium ventricular region in the study case. Hence, integrating DL models into NMI is vital, as it helps to improve image quality and provides alternative solutions that replicate real scans, thereby assisting clinicians in analyzing complex medical data.

### 2.2. Clinical Applications

NMI offers detailed visualization of metabolic processes, which is significant in clinical applications. This section describes the application of NMI in the detection of three types of diseases: neurological disorders, cancers, and cardiovascular disease [36].

#### 2.2.1. Neurology

Most common radiotracers (18F-fluorodeoxyglucose (FDG), 11C-methyl-methionine, etc.) are used to diagnose neurological disorders in NMI processes. However, the high rate of glucose metabolism in normal brain tissue compromises the diagnostic accuracy when FDG is used in low doses. Conversely, targeted amino acid radiotracers (11C-methyl-methionine, 18F-fluoro-l-phenylalanine, 18F-fluoro-L-thymidine, etc.) highlight tumor regions, contribute to grading tumor aggressiveness, and identify tumor recurrence post-surgery. However, 11-C targeted radiotracers are limited by their specific metabolic pathways, which hinders general examination [36]. As a DL-based solution, Kim et al. [37] used a neural network to distinguish between Parkinson’s disease and normal tissue distributions. Despite using a small dataset, which contained 118 abnormal and 63 normal scans, and the retrospective nature of the diagnosis, they achieved high accuracy, with a receiver–operator curve (ROC) value of 0.87.

#### 2.2.2. Oncology

In specific malignancies, such as hepatocellular carcinoma (HCC) in the liver, 18F-FDG is ineffective, with accuracies of only 40–65%. By contrast, PET imaging tracers, such as 11C-acetate (ACT), show a high sensitivity to HCC, being able to detect 78–87% of tumors [38]. Yoo et al. [39] utilized dual-tracer PET/computed tomography (CT) to compare the diagnostic efficacies of 11C-ACT and 18F-FDG in identifying extrahepatic metastases in patients with HCC. Their findings indicated that the tracer avidity of metastatic lesions varied by site; however, comparing tracer avidity was challenging because many patients underwent dual-tracer PET/CT for restaging purposes. Despite these tracers observations, NMI in oncology can be further improved with the use of DL [40].

#### 2.2.3. Cardiology

In cardiology, myocardial perfusion imaging via SPECT or PET scanning is the most common form of diagnosis for patients with coronary artery disease (CAD). Both imaging modalities exhibit limitations due to inaccurate segmentation in the left ventricle (LV) and challenges in identifying myocardial perfusion diseases, which are attributed to low spatial resolution in multi-vessel CAD [41]. Bentancur et al. [42] developed a convolutional neural network to automatically segment the LV myocardium with high accuracy. Otaki et al. [43] developed a DL model to detect CAD in 3578 patients with suspected CAD. The CAD-DL model achieved superior diagnostic accuracy, with an area under the receiver operating characteristic curve (AUC) of 0.83, outperforming the automated quantitative total perfusion deficit (AUC = 0.78) and expert reader diagnosis (AUC = 0.71).

### 2.3. Diffusion and Transformation Learning

**Figure 3:**
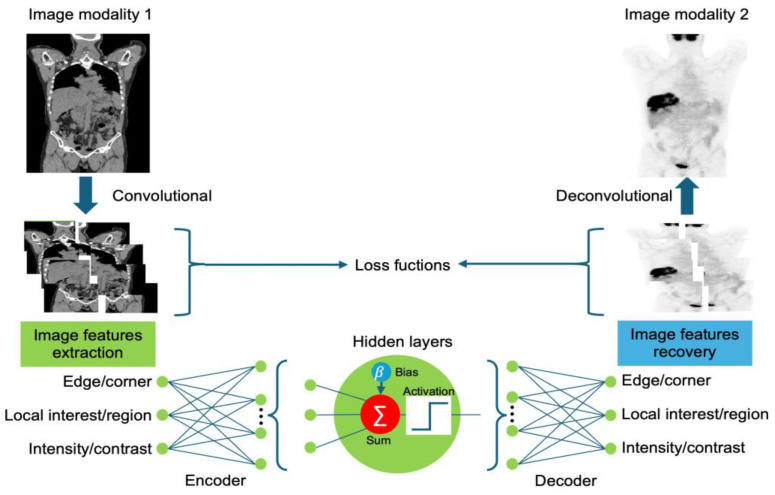
Schematic overview of synthetic image generation in nuclear medicine using DL.

In this subsection, we briefly discuss the use of DL models in NMI. Figure 3 presents a schematic overview of image translation. These learning models leverage convolution and deconvolution networks to extract and recover image features, demonstrating the ability to translate information between different image modalities. A convolution layer, a fundamental component of a convolutional neural network (CNN), utilizes a set of learnable filters to extract meaningful features from input images by transforming image pixels into features [44]. The features are used to examine the input and produce feature maps that emphasize patterns, including edges, textures, and forms. The hidden layers of a neural network can capture complex data patterns. As per the universal approximation theorem, a neural network with a single hidden layer and a nonlinear activation function can approximate any continuous function. However, balancing model complexity with generalization performance is crucial to avoid data overfitting and underfitting. Following each convolution, activation layers perform nonlinear adjustments, allowing the model to capture complex patterns. Common activation functions include rectified linear units (ReLU), to introduce nonlinearity by eliminating negative values, and SoftMax, to convert outputs into probabilities. Depending on the requirements for specific tasks, various activation functions may be used, such as the sigmoid function for binary classification; conversely, no activation function is used for regression. The loss function in DL is used to measure the difference between the predicted image and the real image. This is crucial in model training as it guides the optimization process by reducing the prediction error. Loss functions, such as mean squared error (MSE) and cross-entropy loss, are frequently used for image reconstruction and classification tasks. Finally, deconvolution layers, also referred to as transposed convolution layers, reverse the convolution process, reconstructing low-resolution feature maps back to higher resolutions. Deconvolution layers can be used to upsample and recover features extracted from one image modality and transfer them to other modalities.

#### 2.3.1. Fully Convolutional Neural Network

**Figure 4:**
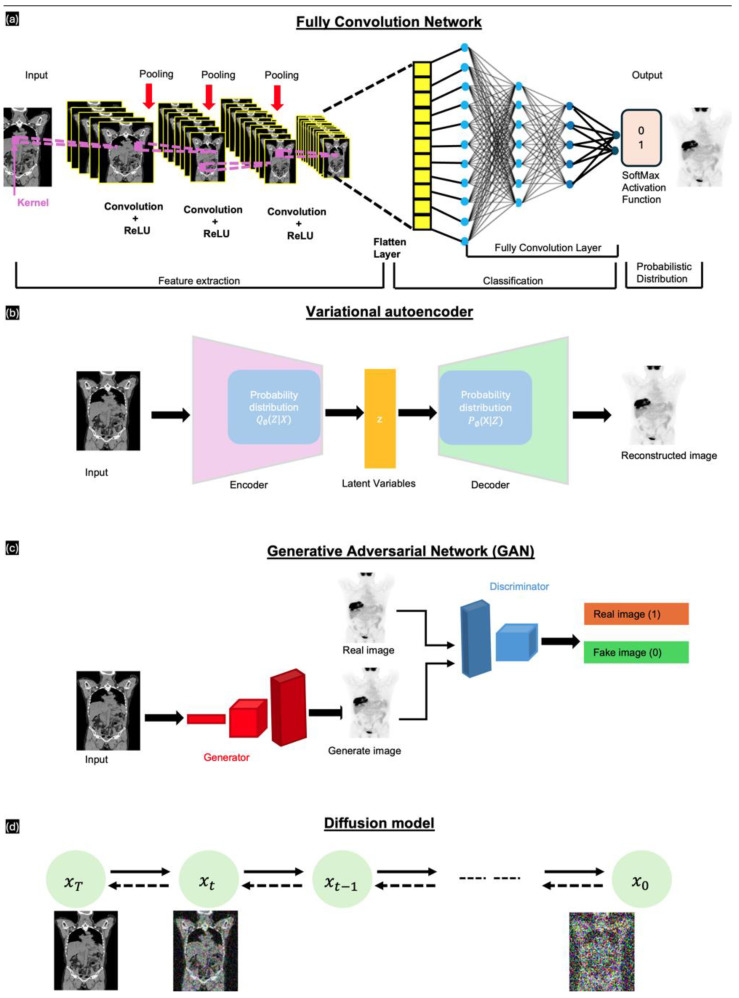
Different general DL models for image synthesis. (**a**) FCNs—convolutional layers, (**b**) VAEs—encode and decode in latent variables, (**c**) GANs—generator and discriminator layers, and (**d**) diffuse model—forwarding and reversing diffusion sequence by adding/removing noise.

Long et al. [45] introduced fully convolutional networks (FCNs); these have been widely used in segmentation tasks, which involve classifying individual pixels into various categories. FCNs are crucial in NMI due to their ability to process medical images of any size, a feature absent in traditional CNNs. This advantage ensures accurate analysis and diagnosis without compromising patient information. As depicted in Figure 4a, an FCN comprises multiple layers arranged sequentially from input to output. These include the ReLU activation layer, followed by pooling layers that downsample the feature maps. After feature extraction, the network flattens the data and employs fully connected layers for classification. The final output uses a SoftMax activation function to generate a probabilistic distribution for classification. The network uses convolution filters or kernels to refine image data, ensure translation invariance, and preserve spatial information. The most common loss functions for FCN-based image-to-image translation include intensity-based pixel-wise loss functions, such as mean absolute error (MAE), which represents the pixel differences between the synthetic image and the ground-truth image.

#### 2.3.2. Variational Autoencoders

Kingma et al. [46] introduced variational autoencoders (VAEs)*,* which have been widely used to generate new data samples that resemble training sets, proving particularly useful in various applications, such as medical imaging. Unlike traditional autoencoders, VAEs are generative models that capture continuous distributions of latent variables. A key advantage of VAEs in medical image synthesis is their ability to learn the complex data distributions in medical images, which enables them to generate realistic synthetic images. However, a common limitation of conventional VAE-based models is the injection of noise or the use of imperfect loss functions, such as L1 or L2 losses, which result in blurry outputs. VAEs comprise two main components, namely an encoder and a decoder. The encoder compresses the input image into a latent space using the probability distribution Q to estimate the latent variable z. These parameters, which define the latent space, enable the generation of new variables that accurately represent the encoder features of the input data. The decoder transforms the latent variable z back into the input, acting as a means of noise distribution. This process is performed using another probability distribution P.

#### 2.3.3. Generative Adversarial Networks

Generative adversarial networks (GANs), introduced by Goodfellow et al. [47], comprise two main subnetworks: a generator and a discriminator (Figure 4c). The generator takes an input originating from a uniform or Gaussian distribution to produce synthetic images that are indistinguishable from the real data. The discriminator receives the generated fake image and determines whether the generated image is fake or real [15]. The generator aims to minimize the probability of the discriminator correctly identifying the generated image as fake. Conversely, the discriminator attempts to maximize its ability to distinguish between the real and generated images. Among the various GAN architectures available, conditional GANs (cGANs; pix2pix [48]) and CycleGAN [49] are significant in medical image synthesis. cGANs generate enhanced medical images using additional information, such as class labels or other modalities. In cGAN-based image generation, magnetic resonance imaging (MRI) data are used as a conditional input to guide the generator in producing a synthetic image. In comparison, CycleGAN uses two sets of generator and discriminator pairs for image translation between two unpaired domains. The two generators translate images between their distribution profiles. The model incorporates a cycle consistency loss, which ensures that images translated from different domains remain indistinguishable from the real images; thus, the translations preserve key features across domains without the need for paired data.

#### 2.3.4. Diffusion

Introduced by Ho et al. [50], the diffusion probabilistic model (DPM) is a novel generative model. The primary goal of diffusion models is to capture the underlying probability distribution of a dataset through a diffusion process. In medical applications, diffusion models are used to generate high-quality synthetic images by learning to reverse the diffusion process. These models are particularly useful for generating detailed and realistic images from noisy inputs and are, therefore, ideal for certain tasks, such as synthetic image generation. Diffusion models function in three key stages—the forward process, the reverse process, and the sampling procedure. The forward process involves incrementally adding Gaussian noise to the data, which progressively transforms the data into pure noise through a series of predefined steps. The data distribution is treated as a Markov chain, where each step is dependent on the previous steps. In contrast, the reverse process—the core process for diffusion models—is focused on denoising the data by learning the probabilities of the reverse Markov chain transitioning from noise to data. During training, this component predicts the noise added at each step, gradually reconstructing the original data from noise. Finally, the sampling procedure reverses the noising process to generate new data samples.

### 2.4. Evaluation

Performance evaluation in NMI-based image synthesis with DL involves various quantitative and qualitative methods to assess the accuracy and quality of the synthesized images. These methods are crucial for validating DL models and ensuring their practicability in clinical settings. We have classified image-related metrics and downstream task decision methods for model comparison, providing a comprehensive evaluation framework that facilitates the optimization of DL models [34].

#### 2.4.1. Image-Related Metrics

The quantitative metrics include MAE, which represents the average pixel-wise difference, and peak signal-to-noise ratio (PSNR), which denotes the maximum possible signal-to-noise ratio; thus, these metrics indicate how closely the synthesized image matches the quality of the reference image. The structure similarity index measure (SSIM) focuses on the similarities between the visible structures of images. Other commonly used evaluation metrics include MSE, root mean squared error (RMSE), Fréchet inception distance, normalized root mean squared error, normalized mean square error, Pearson correlation coefficient, and normalized cross-correlation.

#### 2.4.2. Downstream Task–Related Decisions

While conventional evaluation metrics tend to focus on the visual quality of synthetic images, assessing the synthetic image in downstream tasks, such as segmentation and classification, is also essential for determining clinical usability. By evaluating how well such synthetic images support critical clinical workflow, researchers can gain a better understanding of the real-world applicability and potential benefits of DL models. This approach not only validates the visual accuracy of synthetic images but also their functional utility in various tasks, such as identifying anatomical structures or detecting diseases. In this subsection, we discuss the evaluation of synthetic images in NMI across multiple downstream tasks, emphasizing their usefulness and their impact on clinical practice [34].

Several studies have investigated the practical utility of synthetic images in various clinical applications, emphasizing certain aspects, such as dose distribution assessment and segmentation precision. All NMI reports present a standardized uptake value (SUV) to evaluate the uptake of a radiotracer by finding the ratio between radioactivity measure $c_{img}$ and injected dose ID followed body weight BW, assess metabolic activity differences between benign and malignant lesions (Equation (1)).

(1) $$SUV=\frac{c_{img}}{ID/BW}$$

Chen et al. [51] compared the SUV bias and SUV variance between synthesized PET images and standard full-dose PET images, observing an approximately 8–15% lower SUV bias in the synthesized images. Similarly, Wang et al. [52] evaluated synthesized images based on SUV; they achieved an error margin of 5%, which confirms the clinical reliability of their reconstructions.

Another metric that was used is the dice similarity coefficient (DSC), which evaluates the overlap for assessing the accuracy of segmentation method between the ground truth $S_{gt}$ and predicted sample $S_{p}$ (Equation (2)).

(2) $$DSC=\frac{2|S_{gt}\cap S_{p}|}{\left|S_{gt}\right|+|S_{p}|}$$

Zhuang et al. [53] used DSC to measure the segmentation accuracy of CT images synthesized from T2-weighted MRI scans for the segmentation of eight pelvic structures. They achieved DSC scores of 70.9% and 67.1% on different datasets, respectively. Additionally, Boroojeni et al. [54] demonstrated the effectiveness of synthetic CT images in radiation-free MRI cranial bone imaging, achieving a high DSC of 0.90.

## 3. Synthetic NMI

**Figure 5:**
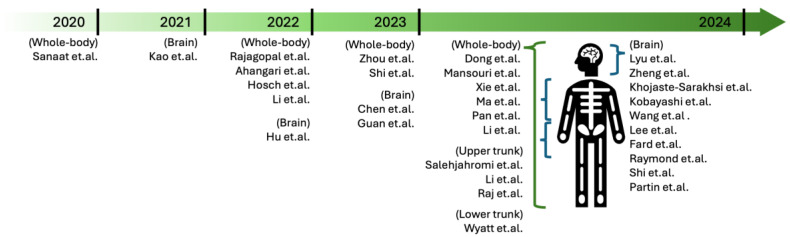
Selected research articles in NMI by publication years and imaging area targets.

In Figure 5, we preset a comprehensive overview of the 31 selected research articles mentioned in Figure 1a by timeline and their imaging area targets (brain, whole-body, upper trunk, or lower trunk). With detailed information, Table 1 lists studies in which MRI and CT images were used to synthesize nuclear medicine images, while Table 2 covers studies in which nuclear medicine images were used as the input. The observations in these tables focus only on aspects related to DL; experimental protocols or design parameters related to data acquisition are not included.

### 3.1. General MRI and CT in NMI

MRI is a powerful tool for medical imaging, utilizing strong magnetic fields and radio waves to generate detailed images of the internal structures of soft tissues [55]. Another imaging method, CT, has revolutionized medical imaging by providing detailed cross-sectional images of internal body structures [56]. In combination with NMI, CT scanning can be performed to construct an attenuation map of the density differences, i.e., attenuation correction (AC), throughout the body, which can then be used to correct for the absorption of photons emitted via isotope decay [57]. When combined with NMI techniques, both MRI and CT imaging provide comprehensive diagnostic information, merging anatomical and functional data [58]. NMI-based image synthesis from general MRI/CT scans involves using advanced computational techniques to generate images that emulate the information provided by nuclear imaging modalities. Various models can be employed to this end, such as CNN, U-Net, or GAN architectures. These models learn to extract features from MRI/CT data that correlate with the functional information provided by PET/SPECT imaging [59].

**Table 1:** Selected MRI/CT-based studies on NMI synthesis.

Ref.	Target	Architecture	Dataset Description	Class
[60]	Translating T1-weighted MRI images to FDG-PET images	U-Net and explainable and simplified image translation	Cognitively normal (300 cases), significant memory concern (54 cases), mild cognitive impairment (868 cases), and Alzheimer’s disease (219 cases)	MRI
[61]	Improving the synthesis of 3D PET images from MRI images	3D unsupervised domain adaptation and 2D s-VAE	146 paired multi-modal MR images from CBICA and 239 paired MR images from TCIA, based on the multi-center BraTS 2019 dataset	MRI
[62]	Generating synthetic whole-body PET images from whole-body MRI data	3D residual U-Net	40 whole-body PET/MRI training exams, 16 whole-body PET/MRI testing exams, and 20 independent pelvic PET/MRI testing exams	MRI
[63]	Generating sCT images from Dixon MRI for whole-body PET-AC	Modified DeepDixon	15 whole-body scans, 11 head-and-neck scans, and 20 thorax and pelvis scans with PET/MRI	MRI
[64]	Generating FDG-PET images from T1-weighted MRI images	Denoising diffusion probabilistic model (DDPM)	1036 FDG-PET/MRI pairs from the Alzheimer’s Disease Neuroimaging Initiative (ADNI)	MRI
[65]	Generating synthetic PET from lung CT scans	Cascaded coarse (fine multi-task)	101 paired imaging data from whole-body sPET	CT
[66]	Generating beta-amyloid PET images from 3D T1-weighted MRI scans	DDPM	180 cognitively normal subjects, 163 early mild cognitive impairment patients, 80 late mild cognitive impairment patients, and MRI/PET scans from the ADNI	MRI
[67]	Creating synthetic PET images of the synaptic vesicle protein 2A (SV2A) from T1-weighted MRI	3D multi-stage (MS) U-Net	54 participants from 22 healthy controls and 32 cases with Alzheimer’s disease.	MRI
[68]	Generating synthetic PET images from 3D MRI scans	3D MS CycleGAN	282 subjects from the ADNI	MRI
[69]	Generating dose map SPECT from CT scans	U-Net transformer	22 patients to generate reference absorbed dose maps via Monte Carlo simulation	CT
[70]	Generating synthetic PET images from CT scans	pix2pix with ResU-Net++	MDA-TRAIN (*n* = 132), MDA-TEST (*n* = 75), TCIA-STANFORD (*n* = 125), LIDC-IDRI (*n* = 655), NSCLC-RT (*n* = 359), and MDA-SCREENING (*n* = 122)	CT
[71]	Generating PET attenuation maps from MRI without CT data	Sim2Real	BrainWeb dataset with 20 MR scans	MRI
[72]	Improving Alzheimer’s disease PET scans by leveraging shared MRI scans	ShareGAN with AdaIN	564 T1-w MRI images and 549 FDG-PET images from ADNI	MRI
[73]	Generating tau PET images from other types of neuroimaging data	3D dense U-Net	T1w, FDG-PET, amyloid-PET, and tau-PET (*n* = 1192, number of scans = 1505)	MRI

**Figure 6:**
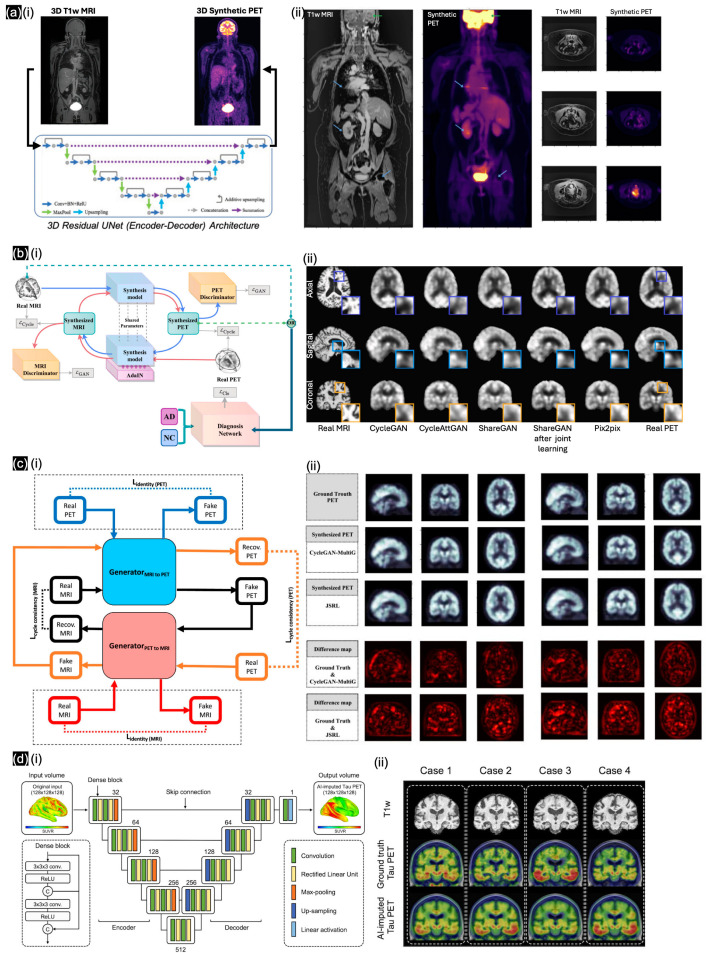
(**a**) (**i**) 3D residual U-Net architecture for generating synthetic PET images from MRI images, exhibiting significant stitching artifacts (blue arrows) in (**ii**) T1w-MRI between bed positions as well as loss in resolution in the head (green arrows); with different cross-sections denoted by blue arrows. Reproduced with permission from Rajagopal et al. [62]; published by IEEE Xplore, 2022. (**b**) (**i**) Overview of CycleGAN framework for MRI-to-PET translation, and (**ii**) visualization of ground-truth and synthetic PETs. Reproduced with permission from Khojaste-Sarakhsi et al. [68]; published by Image and Vision Computing—ScienceDirect, 2024. (**c**) (**i**) Novel joint learning framework combining unsupervised cross-modal synthesis and diagnosis for Alzheimer’s disease by mining underlying shared modality information to improve performance. Qualitative results of (**ii**) different cross-modal synthesis networks with SUV ratio error map between real PET image and synthesized PET image. Reproduced with permission from Wang et al. [72]; published by Medical Image Analysis—ScienceDirect, 2024. (**d**) (**i**) Dense U-Net architecture, and (**ii**) FDG-PET based tau-PET synthesis result. Reproduced with permission from Lee et al. [73]; published by Brain—Oxford Academic, 2024.

The first publication we identified regarding general MRI in NMI was by Kao et al. [60]. When U-Net was introduced in 2015, directly applying the image translation model from T1w-MRI to synthetic FDG-PET was not feasible. Through the incorporation of explainable and simplified image translation, four distinct patterns of regional hypermetabolism and hypometabolism were identified from the Alzheimer’s Disease Neuroimaging Initiative dataset (ADNI), which substantiates our hypothesis regarding the region-dependent transformation necessary for inferring final PET images. This approach facilitates the understanding of regional metabolic differences, including normal variations, age-related changes, and dementia-associated alterations, with canonical component analysis similarities of up to 0.75. To address the complexity of 3D volumetric samples, Hu et al. [61] introduced unsupervised domain adaptation (UDA) for 3D medical image synthesis and proposed an efficient 2D VAE approach to perform 3D UDA with spatial VAE for dimensionality reduction, achieving an SSIM of over 0.838. In the same year, Rajagopal et al. [62] trained the FCNs in a 3D residual U-Net (Figure 6(ai)) on data from 40 whole-body PET/MRI exams. The quantitative results from Rajagopal’s model followed, with visualization in cross-section images (Figure 6(aii)) by absolute error (MAE) and predicted quantification error as the lowest values (≤7.6%) for various real and synthetic PET data sources. Ahangari et al. [63] introduced a modified DeepDixon CNN with a 3D U-Net, which was pretrained from 811 previous brain scans and then applied to other regional data, including the thorax, pelvis, and head-and-neck regions. Their accuracy and feasibility were compared and showed a 5.7% improved compared to the conventional atlas-based method for whole-body PET/MRI and more accurate estimation of tracer uptake, as shown by smaller standard deviations of errors. Chen et al. [64] developed a pipeline for synthesizing FDG-PET/MRI images from source MRI-T1w images based on the denoising diffusion probabilistic model (DDPM); they validated the effectiveness of the synthesized FDG-PET images in terms of accuracy and F1-score on 1036 FDG-PET/MRI pairs from the ADNI dataset. Lyu et al. [66] showed that T1-w MRI scans, which have a different function from beta-amyloid PET scans, could be used as an alternative input for diagnosing Alzheimer’s disease; based on data from 180 cognitively normal subjects, 163 early mild cognitive impairment patients, and 80 late mild cognitive impairment patients, they achieved a high SSIM (up to 0.911 ± 0.014) using the modified DDPM. With the same target, Khojaste-Sarakhsi et al. [68] developed an unsupervised learning framework with a 3D multi-scale CycleGAN for generating synthetic PET images from MRI images (Figure 6(bi)), using the data of only 282 subjects from the ADNI dataset. A comparison of the synthetic and ground-truth PET images (Figure 6(bii)) highlighted the potential for generating synthetic images with similar quality (SSIM up to ~0.8). With a specific biomarker in Alzheimer’s disease, Zheng et al. [67] implemented a 3D multi-stage (MS) U-Net to generate specific 11C-UCB-J PET images and to find the associated information with SV2A-PET/MRI images. Trained on the clinical MRI/PET data of 160 subjects, the model could generate PET images highly similar to the ground-truth images (SSIM of up to 0.90 ± 0.05), with a low injection dose (<5 mCi) as the clinical protocol. In a similar effort to reduce the radiation dose for patients, Kobayashi et al. [71] performed AC without CT using a simulated PET dataset of the human brain based on only 20 MRI brain scans from the BrainWeb dataset. Finally, Wang et al. [72] presented an unsupervised cross-modal synthesis network, ShareGAN, which utilizes the AdaIN module to enable interconversion between 3D PET and MRI images (Figure 6(ci)); this approach diversifies the modality information by leveraging 564 T1-w MRI images and 549 FDG-PET images from the ADNI. Based on cross-section visualizations (Figure 6(cii)), ShareGAN, trained via joint learning, exhibited a higher SSIM (up to 0.916) than previously developed GAN frameworks. With the largest collection of multi-modality databases (1192 unique individuals), Lee et al. [73] developed a 3D dense U-Net model (Figure 6(di)) that enables cross-modal tau-PET synthesis from different types of neuroimaging data, including FDG-PET, structural T1-w, and amyloid PET images (Figure 6(dii)). Based on comparisons with multiple popular learning models, such as VAE and pix2pix, the optimized 3D dense U-Net was found to perform best (in terms of Pearson’s correlation) when trained on a large dataset.

**Figure 7:**
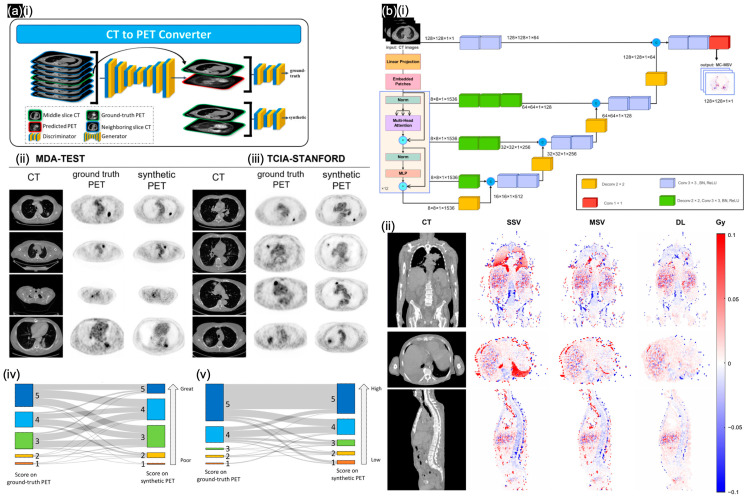
(**a**) (**i**) cGAN to synthesize PET images from CT scans. Validation of synthetic PET images based on (**ii**) MDA-TEST and (**iii**) TCIA-STANFORD testing cohorts; (**iv**) imaging quality difference; (**v**) tumor contrast difference. Reproduced with permission from Salehjahromi et al. [70]; published by Cell Reports Medicine—Elsevier, 2024. (**b**) (**i**) UNETR architecture to generate MS-based dose maps. (**ii**) Relative absolute error maps in cross-section views of SSV/MSV/DL methods. Reproduced with permission from Mansouri et al. [69]; published by EJNMMI—Springer Nature, 2024.

Regarding general CT in NMI, Dong et al. [65] employed cascaded coarse and fine multi-task models for the segmentation of contours associated with abnormal metabolic activity; this approach facilitates the simultaneous generation of whole-body PET images and tumor-region images, followed by the acquisition of the sPET images through the fusion of the 101 pairs of imaging data from whole-body sPET scans. Salehjahromi et al. [70] improved the GAN-based framework to generate high-fidelity synthetic PET images from CT scans (Figure 7a). The synthetic PET images were validated by thoracic radiologists and through radio genomics analysis; these images demonstrated high fidelity and biological accuracy compared with actual PET scans, based on the MDA-TEST and TCIA-STANDFORD datasets (Figure 7(aii,aiii)). A Turing test was conducted, in which the quality of the synthetic PET images was subjectively rated on a 5-point scale (Figure 7(aiv)). Based on both the mean imaging quality ratings (Figure 7(av)) and the identification of the synthetic scans in the Turing test, the radiologists achieved an overall 75% accuracy and misclassified 7% of the synthetic cases with high lesion contrast evaluation (99%). Through specific radiopharmaceutical therapy, Mansouri et al. [69] developed a hybrid transformer-based DL model called U-Net transformer (UNETR), which incorporates a CT voxel approach for voxel-level dosimetry (Figure 7(bi)). This model achieved better performance when compared in time efficiency and a higher gamma analysis pass rate (up to 99%) with a similar dose to simulation results with formalisms (MSV/SSV) (Figure 7(bii)).

### 3.2. NMI Translation

**Figure 8:**
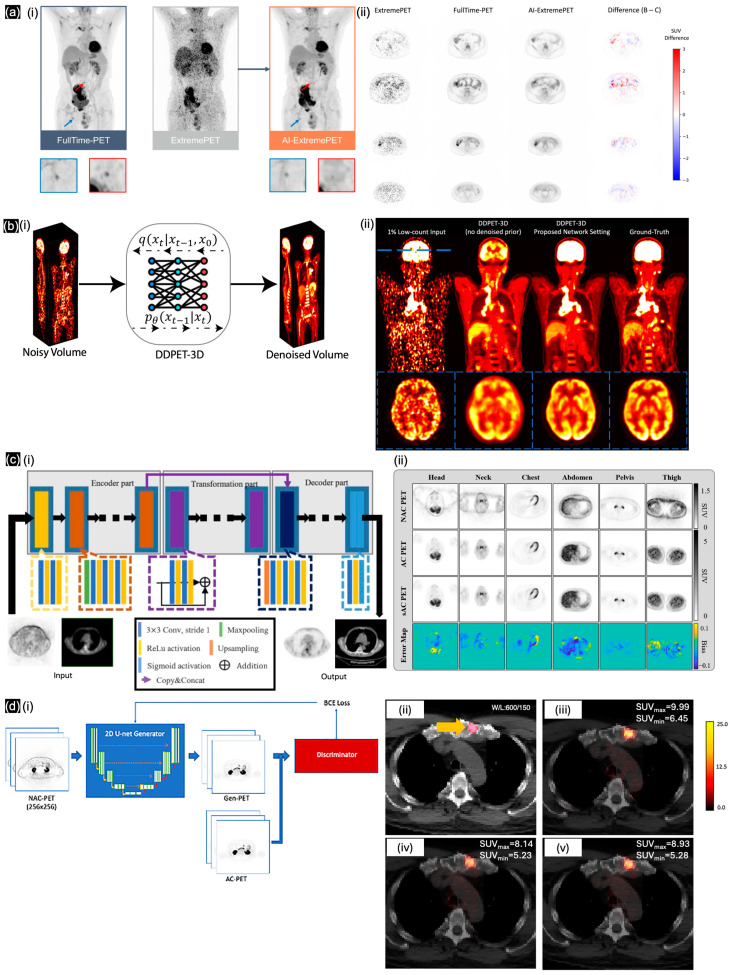
(**a**) (**i**) pix2pixHD to generate AI-ExtremePET from ExtremePET. (**ii**) Comparison between different cross-sections. Reproduced with permission from Hosch et al. [75]; published by EJNMMI—Springer, 2022. (**b**) (**i**) DDPET-3D model to generate synthetic full-dose PET volume from a low-count PET volume. (**ii**) Represents the whole-body/brain’s cross-section between synthetic and ground-truth PETs. Reproduced with permission from Xie et al. [76]; published by AxRiv, 2024. (**c**) (**i**) Modified pix2pix model and (**ii**) comparison of representative PET images in specific regions with error map. Reproduced with permission from Li et al. [77]; published by the European Journal of Radiology—ScienceDirect, 2022. (**d**) (**i**) Synthetic PET images produced by training a 2D pix2pix model, overlaid on CT and compared with (**ii**) original CT, (**iii**) original AC-PET on CT, (**iv**) V1-PET on CT, and (**v**) V2-PET on CT. Reproduced with permission from Ma et al. [78]; published by Oncotarget—Impact Journals, 2024.

Nuclear medicine is used to elucidate medical conditions in patients through an examination of how the body receives certain radiation markers. Image translation in NMI offers several significant benefits, such as enhanced image quality from low-dose imaging and personalized dosimetry with AC [74]. Figure 8 shows low-dose images generated using different methods.

To enhance image quality under low-dose imaging, Sanaat et al. [79] used DL algorithms to synthesize accurate low-dose whole-body PET images from images acquired under a noticeably reduced radiotracer dose and scan duration. They trained CycleGAN on the data of 100 patients who underwent full-dose PET with a ~7-fold faster scan than standard. The synthetic PET images produced by the pretrained CycleGAN exhibited higher quality than low-dose scan images while attaining similar quality to full-dose PET images. Hosch et al. [75] trained a cGAN (pix2pixHD) (Figure 8(ai)) with 587 datasets to enhance an ultra-low-count FDG PET scan with under 30 s of whole-body acquisition time. Hosch’s model presented the similarity in cross-section of the SUV difference with an acceptable value (under 1.5) and enhanced patient-based sensitivity with specificity for lesion detection up to 79% (Figure 8(aii)). Presenting the federated transfer learning method, Zhou et al. [80] mentioned the necessity when acquiring a lower amount of representative data for training while addressing the large domain shift caused by low-dose PET denoising using heterogeneous low-dose data. As a solution, Zhou trained the dual-attention residual dense U-Net (DuAttRDUNet) as a PET denoising network from 175 whole-body subjects in only 20%-dose PET imaging. Zhou provided a similarity SSIM value of 0.978 for their synthetic PET denoising, close to full-dose PET imaging. Fard et al. [81] developed a multi-channel MRI/PET GAN framework to generate cross-modality synthetic SPECT images based on 48 brain scans. This model achieved a 5.4% higher SSIM compared with original SPECT images under lower radiation exposure. Raymond et al. [82] developed the SMART-PET framework, which leverages self-similarity awareness for denoising FDG-PET images, enabling a 90% dose reduction based on 114 human brain data samples. To understand the relation between imaging details from multiple sources, Shi et al. [83] designed a privacy-enhanced latent diffusion model (PE-LDM) model to generate PET images from MRI or CT images as well as enhance PET images using the super-resolution technique. Pan et al. [84] trained a PET consistency model (PET-CM) based on DPM using data from 35 patients containing 11,200 slices from full-dose to quarter-dose to estimate full-dose PET images; this model improves image quality while reducing the dose requirement by 75%. Finally, Xie et al. [76] proposed the DDPET-3D model (Figure 8(bi)), which utilizes multiple neighboring low-count PET slices as additional inputs to predict a synthetic central PET slice, and this model was trained on real low-dose datasets containing less than 50% of full-dose whole-body PET data from four data centers (Figure 8(bii)).

To personalize dosimetry with AC and without CT scans, Li et al. [77] generated PET attenuation maps and non-AC PET (NAC-PET) pseudo-CT images from 34 lymphoma patients using the pix2pix model (Figure 8(ci)); the cross-sections were visualized in SUV difference maps (Figure 8(cii)). Guan et al. [85] used synthetic CT generation via a variant invertible network (IVNAC) to directly predict attenuation-corrected PET images with a multi-component loss function. By training the IVNAC model using head PET/CT scan data from 37 patients, they achieved the best RMSE among previously developed learning models (up to 1.23%). Shi et al. [86] proposed the DeepImage-PET model, which utilizes an extra physics-based loss function to enhance the accuracy of PET attenuation physics. This model can generate CT-based attenuation maps through the simultaneous reconstruction of PET activity and attenuation of low-dose oncological PET using an image-domain loss function (IM-loss). In both full-dose and low-dose experiments, the suggested framework attained an error rate of less than 1% in tumor-standardized absorption value measurements. Ma et al. [78] presented a modified pix2pix model (Figure 8(di)) that utilizes paired AC-PET and NAC-PET data derived from 302 patients with prostate cancer. The SUVs of the synthetic AC-PET images were highly similar to those of the original images, with a correlation coefficient of up to 0.89 (Figure 8(dii)). Li et al. [87] investigated the production of CT-free AC-PET using CycleGAN with data from 122 whole-body patient scans conducted at a private data center. The CycleGAN model, trained with four loss functions, demonstrated a 35% enhancement in SSIM for NAC PET images. Li et al. [88] employed three learning models, each incorporating multiple loss functions with a U-Net architecture, to generate AC-PET images from breast PET/MRI data that initially yielded only NAC-PET. An SUV assessment indicated no substantial difference between MSE loss and perceptual loss when using a pretrained U-Net to compare synthetic AC-PET images with Dixon-based sCT images. Wyatt et al. [89] employed a previously established learning model for MRI/CT to reutilize and transfer their pretrained multi-task U-Net framework, which incorporates various loss functions specifically for image translation and bone density value estimation; this model was utilized for synthetic CT in attenuation correlation. Focusing on the brain, Partin et al. [90] explored the use of DL for AC in brain FDG-PET imaging without relying on CT scans. With the aim of reducing radiation exposure, the 3D U-Net provided an attenuation map and confirmed the effect of air cavities and facial bones on synthetic CT in non-AC PET, which is ignored in commercial analytical methods, such as Smart Neuro AC (SNAC). Statistical analysis confirmed significant improvements over commercial methods, with the DL method and the commercial method achieving precision levels of 0.92 and 0.78, respectively.

**Table 2:** Selected nuclear medicine studies using nuclear medicine images as input.

Ref.	Target	Architecture	Dataset Description	Class
[79]	Generating full-dose PET images from low-dose PET	CycleGAN	100 patients who underwent F-FDG PET/CG scans	PET enhancing
[77]	Generating PET attenuation maps and pseudo-CT images from NAC PET images	pix2pix	34 lymphoma patients who underwent whole-body PET/CT imaging	Synthetic AC
[75]	Reducing PET acquisition times while maintaining diagnostic quality	Modified pix2pixHD	587 PET/CT scans in full-dose or low-dose technique	PET enhancing
[80]	Improving the quality of low-dose PET images	DuAttRDUNet with FTL	175 subjects with heterogeneous low-dose PET/CT	PET enhancing
[85]	Generating sCT images for brain PET-AC from NAC-PET images	IVNAC	Head PET/CT scans of 37 patients	Synthetic AC
[86]	Generating accurate PET attenuation maps	DeepImage-PET	100 skull-to-toe FDG-PET/CT scans	Synthetic AC
[81]	Generating interictal SPECT images from MRI and PET scans	pix2pix	Standard PET, SPECT, and MPRAGE T1-w MRI images from 86 subjects	PET enhancing
[82]	Denoising PET images by leveraging self-similarity	SMART-PET	114 human brain data samples from six PET/MRI studies	PET enhancing
[78]	Generating AC-PET images using NAC-PET	pix2pix	183 training, 60 validation, and 59 independent testing studies	Synthetic AC
[91]	Recovering spatially variant deformations in dual-panel PET	3D U-Net	70 pairs of reconstructed dual-panel breast PET systems (B-PET)	PET enhancing
[87]	Generating AC-PET images without CT scans	CycleGAN	Whole-body PET data from 122 subjects (29 females and 93 males)	Synthetic AC
[83]	Enhancing synthetic PET images from multiple sources	PE-LDM	Mayo low-dose CT dataset and IXI brain MRI dataset: 2377 CT, 7000 MRI, and 7000 PET synthetic images	PET enhancing
[88]	Enhancing AC-PET with synthetic sCT images from NAC-PET/MRI images	Multiple-loss U-Net	PET/CT and PET/MR scans of 23 female subjects with invasive breast cancer	Synthetic AC
[89]	Enhancing the accuracy of AC-PET/MRI by generating sCT images	Multi-task U-Net	ZTE and CT scans of 36 pelvic radiotherapy patients	Synthetic AC
[84]	Generating full-dose PET images from low-dose PET scans	PET-CM	11,200 slices across 35 patients, from full-dose to quarter-dose	PET enhancing
[90]	DL-based CT-less AC of brain FDG PET	3D U-Net	100 FDG PET-CT brain images of adults with suspected dementia	Synthetic AC
[76]	Generating and denoising 3D PET images from low-count PET images	DDPET-3D	5933 images from 1167 patients	PET enhancing

## 4. Discussion

The combination of NMI with other medical imaging modalities, regardless of the difference in their uses, represents an effective approach for both statistical population diagnosis and individualized treatment for patients. Modern DL methodologies for image synthesis are enhancing the practical applicability of NMI in healthcare, in line with proven gold-standard policies [3]. Image synthesis in NMI could be considered to extract hidden features from MRI or CT scans, which are hardly recognizable without actual NMI processing. Image synthesis in NMI proved the relation between medical imaging modalities in the mentioned studies. Some studies improved the AC of NMI by generating CT images from CT-less systems, demonstrating that NAC-PET systems can self-correct for attenuation. Using synthetic nuclear medicine images, radiologists can determine whether a patient requires NMI as well as estimate the safe dose limit with minimal side-effects. NMI-based image synthesis can be used to find similarities between an individual patient’s data and diagnostic patterns observed in population group.

In this review, we examined 4 general deep learning models—FCNs, VAEs, GANs, and diffusion models, along with their variants—by presenting 31 publications. Table 1 and Table 2 showed the high variability of GANs and diffusion models, which is attributed to the limitations of clinical datasets. These challenges could be addressed through iteration learning with feedback layers, such as discriminators in GANs and reversing loops in diffusion models. Most U-Net variants (FCNs) were utilized without feedback layers due to their lower memory requirements during training, making them suitable for handling large datasets. No publications involved VAEs, likely due to the limited clinical interpretability of their latent representations and their high computational demands [61]. To overcome these specific limitations, recent research predominantly focuses on cross-modality learning models, such as 3D supervised domain adaptation and 2D s-VAE [62], pix2pix and ResU-Net++ [71], multi-task U-Net [90], and multiple-loss U-Net approaches. Additionally, physically driven learning methods, such as those discussed in [72], have been proposed as an efficient learning method with physics simulation.

Another significant gap in the practical implications of image synthesis in NMI is the challenge of establishing trust in AI-based diagnostics. First, many studies rely predominantly on local NMI datasets, which complicates the replication and validation of their theoretical conclusions due to data privacy regulations that restrict access to public databases (e.g., the ADNI database, Kaggle libraries). The lack of theoretical investigations further impedes the assessment of NMI’s broader applicability. Second, these studies did not address how NMI-based diagnostics account for variability in clinical performance metrics (e.g., ROC analysis, SUV, and scoring). Instead, all studies reviewed herein used general evaluation methods in computer vision (MSE, MAE, SNR, PSNR, and SSIM) as the initial step to assess the quality of DL models. Third, the focus on single-target image translation models (e.g., brain, upper/lower trunk) aligned with specialized medical investigations but limits our ability to understand systemic interactions or correlations between different body regions. While “whole-body” NMI translation models provide a broader perspective and facilitate technical substitutions (e.g., AC or image enhancement), no studies to date have provided specific clinical analyses addressing the relationships between different body regions (e.g., inter-organ or brain–organ correlations).

In the future, the challenges associated with building trust in AI-based diagnostics are expected to diminish due to several developments. First, emerging research is increasingly required to adhere to systematic design protocols, standardized reporting guidelines, and explicit definitions of training, validation, and testing datasets [92]. Although the limited availability of specific NMI datasets poses challenges for improving image synthesis through DL, the integration of public databases offers opportunities for AI developers to identify relationships and validate the performance of learning models across both public and specialized NMI datasets. Second, the limitations of NMI-based diagnostics may be addressed through the adoption of large language models (LLMs), which hold the potential to support the analysis of NMI data [93]. LLMs can provide valuable information by filling in missing labels and assisting in the classification of radiological findings based on clinical diagnostic metrics (e.g., SUV, scoring systems). Third, public datasets of annotated regions and organs [94,95] need to carefully review and integrated with translation models and clinical trial reports (e.g., the metastasizing mechanisms of lung cancer [96]). These advancements collectively aim to enhance the reliability and applicability of AI in NMI diagnostics.

## 5. Conclusions

To develop a comprehensive approach for both population-level diagnosis and personalized treatment, one must combine NMI with other medical imaging modalities. Utilizing NMI-based image synthesis enhances diagnostic accuracy and enables the establishment of standard protocols. Additionally, researchers have developed current image synthesis methods (FCNs, VAEs, GANs, and diffusion models) to assist clinicians in segmentation and provide self-correction from MRI or CT-less scans for PET system attenuation. While NMI translation is a unique topic, current research can aid in image translation for AC maps, enabling radiologists to estimate appropriate doses or assess the need for nuclear medicine procedures. In this review, cross-modality learning models or simulation-driven learning support were used to improve the problems with image synthesis deep learning methods. Issues with establishing trust in AI-based diagnostics surfaced when the comprehension of their practical implications was incomplete. We should determine the reporting standards and systematic design for clinical datasets and share them under public licenses. Another DL generator should be applied to account for the missing information or generate the radiology report, such as an LLM or image realistic generation. After a thorough review of public datasets and clinical trial reports, the DL model should establish a connection between various body functions and various tracer targets. Addressing these gaps, future research should concentrate on integrating clinical performance metrics to harmonize these advancements with transformation learning in clinical practices and enhance decision-making, despite the widespread use of traditional evaluation metrics.

## References

[1] Könik A., O’Donoghue J.A., Wahl R.L., Graham M.M., Van den Abbeele A.D. (2021). Theranostics: The Role of Quantitative Nuclear Medicine Imaging. Semin. Radiat. Oncol..

[2] Wahl R.L. (1998). Progress in Nuclear Medicine Imaging of Cancers. Prim. Care Clin. Off. Pract..

[3] Le D., Wong F.C.L. (2021). An Overview of the Regulations of Radiopharmaceuticals. Locoregional Radionuclide Cancer Therapy: Clinical and Scientific Aspects.

[4] Mariani G., Bruselli L., Kuwert T., Kim E.E., Flotats A., Israel O., Dondi M., Watanabe N. (2010). A Review on the Clinical Uses of SPECT/CT. Eur. J. Nucl. Med. Mol. Imaging.

[5] Townsend D.W., Carney J.P.J., Yap J.T., Hall N.C. (2004). PET/CT Today and Tomorrow. J. Nucl. Med..

[6] Ge J., Zhang Q., Zeng J., Gu Z., Gao M. (2020). Radiolabeling Nanomaterials for Multimodality Imaging: New Insights into Nuclear Medicine and Cancer Diagnosis. Biomaterials.

[7] Eary J.F. (1999). Nuclear Medicine in Cancer Diagnosis. Lancet.

[8] Kircher M., Lapa C. (2017). Novel Noninvasive Nuclear Medicine Imaging Techniques for Cardiac Inflammation. Curr. Cardiovasc. Imaging Rep..

[9] Ouvrard E., Kaseb A., Poterszman N., Porot C., Somme F., Imperiale A. (2024). Nuclear Medicine Imaging for Bone Metastases Assessment: What Else besides Bone Scintigraphy in the Era of Personalized Medicine?. Front. Med..

[10] Love C., Palestro C.J. (2016). Nuclear Medicine Imaging of Bone Infections. Clin. Radiol..

[11] Mullan B.P. (2004). Nuclear Medicine Imaging of the Parathyroid. Otolaryngol. Clin. N. Am..

[12] Skoura E. (2013). Depicting Medullary Thyroid Cancer Recurrence: The Past and the Future of Nuclear Medicine Imaging. Int. J. Endocrinol. Metab..

[13] Hilson A.J.W. (2003). Functional Renal Imaging with Nuclear Medicine. Abdom. Imaging.

[14] Kusmirek J.E., Magnusson J.D., Perlman S.B. (2020). Current Applications for Nuclear Medicine Imaging in Pulmonary Disease. Curr. Pulmonol. Rep..

[15] Bennink R.J., Tulchinsky M., de Graaf W., Kadry Z., van Gulik T.M. (2012). Liver Function Testing with Nuclear Medicine Techniques Is Coming of Age. Semin. Nucl. Med..

[16] Toney L.K., McCue T.J., Minoshima S., Lewis D.H. (2011). Nuclear Medicine Imaging in Dementia: A Practical Overview for Hospitalists. Hosp. Pract..

[17] Aghakhanyan G., Di Salle G., Fanni S.C., Francischello R., Cioni D., Cosottini M., Volterrani D., Neri E. (2023). Radiomics Insight into the Neurodegenerative “Hot” Brain: A Narrative Review from the Nuclear Medicine Perspective. Front. Nucl. Med..

[18] Rostami M., Oussalah M., Berahmand K., Farrahi V. (2023). Community Detection Algorithms in Healthcare Applications: A Systematic Review. IEEE Access.

[19] Arnaud M., Bégaud B., Thurin N., Moore N., Pariente A., Salvo F. (2017). Methods for Safety Signal Detection in Healthcare Databases: A Literature Review. Expert. Opin. Drug Saf..

[20] Le T.D., Kwon S.-Y., Lee C. (2022). Segmentation and Quantitative Analysis of Photoacoustic Imaging: A Review. Photonics.

[21] Liu C., Amodio M., Shen L.L., Gao F., Avesta A., Aneja S., Wang J.C., Priore L.V.D., Krishnaswamy S. CUTS: A Deep Learning and Topological Framework for Multigranular Unsupervised Medical Image Segmentation 2024. Proceedings of the International Conference on Medical Image Computing and Computer-Assisted Intervention.

[22] Son J., Park S.J., Jung K.-H.H. (2017). Retinal Vessel Segmentation in Fundoscopic Images with Generative Adversarial Networks. arXiv.

[23] Pinnock R., Ritchie D., Gallagher S., Henning M.A., Webster C.S. (2021). The Efficacy of Mindful Practice in Improving Diagnosis in Healthcare: A Systematic Review and Evidence Synthesis. Adv. Health Sci. Educ..

[24] Brown S., Castelli M., Hunter D.J., Erskine J., Vedsted P., Foot C., Rubin G. (2014). How Might Healthcare Systems Influence Speed of Cancer Diagnosis: A Narrative Review. Soc. Sci. Med..

[25] Mohd Sagheer S.V., George S.N. (2020). A Review on Medical Image Denoising Algorithms. Biomed. Signal Process. Control.

[26] Kaur S., Singla J., Nikita, Singh A. Review on Medical Image Denoising Techniques. Proceedings of the 2021 International Conference on Innovative Practices in Technology and Management (ICIPTM).

[27] Mroueh N., Parakh A., Serrao J., Lee S.I., Eisner B.H., Gervais D.A., Kambadakone A.R., Sahani D.V. (2023). The Why, Who, How, and What of Communicating CT Radiation Risks to Patients and Healthcare Providers. Abdom. Radiol..

[28] Gupta S.K., Ya’qoub L., Wimmer A.P., Fisher S., Saeed I.M. (2020). Safety and Clinical Impact of MRI in Patients with Non–MRI-Conditional Cardiac Devices. Radiol. Cardiothorac. Imaging.

[29] Oglevee C., Pianykh O. (2015). Losing Images in Digital Radiology: More than You Think. J. Digit. Imaging.

[30] Xia Y., Zhang L., Ravikumar N., Attar R., Piechnik S.K., Neubauer S., Petersen S.E., Frangi A.F. (2021). Recovering from Missing Data in Population Imaging—Cardiac MR Image Imputation via Conditional Generative Adversarial Nets. Med. Image Anal..

[31] Raad R., Ray D., Varghese B., Hwang D., Gill I., Duddalwar V., Oberai A.A. (2024). Conditional Generative Learning for Medical Image Imputation. Sci. Rep..

[32] Yang H.S., Rhoads D.D., Sepulveda J., Zang C., Chadburn A., Wang F. (2022). Building the Model: Challenges and Considerations of Developing and Implementing Machine Learning Tools for Clinical Laboratory Medicine Practice. Arch. Pathol. Lab. Med..

[33] Visvikis D., Cheze Le Rest C., Jaouen V., Hatt M. (2019). Artificial Intelligence, Machine (Deep) Learning and Radio(Geno)Mics: Definitions and Nuclear Medicine Imaging Applications. Eur. J. Nucl. Med. Mol. Imaging.

[34] Dayarathna S., Islam K.T., Uribe S., Yang G., Hayat M., Chen Z. (2024). Deep Learning Based Synthesis of MRI, CT and PET: Review and Analysis. Med. Image Anal..

[35] Giammarile F., Knoll P., Kunikowska J., Paez D., Estrada Lobato E., Mikhail-Lette M., Wahl R., Holmberg O., Abdel-Wahab M., Scott A.M. (2024). Guardians of Precision: Advancing Radiation Protection, Safety, and Quality Systems in Nuclear Medicine. Eur. J. Nucl. Med. Mol. Imaging.

[36] Visvikis D., Lambin P., Beuschau Mauridsen K., Hustinx R., Lassmann M., Rischpler C., Shi K., Pruim J. (2022). Application of Artificial Intelligence in Nuclear Medicine and Molecular Imaging: A Review of Current Status and Future Perspectives for Clinical Translation. Eur. J. Nucl. Med. Mol. Imaging.

[37] Kim D.H., Wit H., Thurston M. (2018). Artificial Intelligence in the Diagnosis of Parkinson’s Disease from Ioflupane-123 Single-Photon Emission Computed Tomography Dopamine Transporter Scans Using Transfer Learning. Nucl. Med. Commun..

[38] Salem N., Kuang Y., Corn D., Erokwu B., Kolthammer J.A., Tian H., Wu C., Wang F., Wang Y., Lee Z. (2011). [(Methyl)1-11C]-Acetate Metabolism in Hepatocellular Carcinoma. Mol. Imaging Biol..

[39] Yoo S.W., Kim D.-Y., Pyo A., Jeon S., Kim J., Kang S.-R., Cho S.-G., Lee C., Kim G.-J., Song H.-C. (2021). Differences in Diagnostic Impact of Dual-Tracer PET/Computed Tomography According to the Extrahepatic Metastatic Site in Patients with Hepatocellular Carcinoma. Nucl. Med. Commun..

[40] Hirata K., Sugimori H., Fujima N., Toyonaga T., Kudo K. (2022). Artificial Intelligence for Nuclear Medicine in Oncology. Ann. Nucl. Med..

[41] Bateman T.M. (2012). Advantages and Disadvantages of PET and SPECT in a Busy Clinical Practice. J. Nucl. Cardiol..

[42] Betancur J., Rubeaux M., Fuchs T., Otaki Y., Arnson Y., Slipczuk L., Benz D., Germano G., Dey D., Lin C.-J. (2016). Automatic Valve Plane Localization in Myocardial Perfusion SPECT/CT by Machine Learning: Anatomical and Clinical Validation. J. Nucl. Med..

[43] Otaki Y., Singh A., Kavanagh P., Miller R.J.H., Parekh T., Tamarappoo B.K., Sharir T., Einstein A.J., Fish M.B., Ruddy T.D. (2022). Clinical Deployment of Explainable Artificial Intelligence of SPECT for Diagnosis of Coronary Artery Disease. JACC Cardiovasc. Imaging.

[44] Currie G., Rohren E. (2021). Intelligent Imaging in Nuclear Medicine: The Principles of Artificial Intelligence, Machine Learning and Deep Learning. Semin. Nucl. Med..

[45] Long J., Shelhamer E., Darrell T. Fully Convolutional Networks for Semantic Segmentation 2015. Proceedings of the Proceedings of the IEEE Conference on Computer Vision and Pattern Recognition.

[46] Kingma D.P., Welling M. (2022). Auto-Encoding Variational Bayes 2022. arXiv.

[47] Goodfellow I., Pouget-Abadie J., Mirza M., Xu B., Warde-Farley D., Ozair S., Courville A., Bengio Y. (2020). Generative Adversarial Networks. Commun. ACM.

[48] Isola P., Zhu J.-Y., Zhou T., Efros A.A. Image-to-Image Translation with Conditional Adversarial Networks 2018. Proceedings of the Proceedings of the IEEE Conference on Computer Vision and Pattern Recognition.

[49] Zhu J.-Y., Park T., Isola P., Efros A.A. Unpaired Image-to-Image Translation Using Cycle-Consistent Adversarial Networks. Proceedings of the 2017 IEEE International Conference on Computer Vision (ICCV).

[50] Ho J., Jain A., Abbeel P. (2020). Denoising Diffusion Probabilistic Models 2020. arXiv.

[51] Chen K.T., Gong E., de Carvalho Macruz F.B., Xu J., Boumis A., Khalighi M., Poston K.L., Sha S.J., Greicius M.D., Mormino E. (2019). Ultra–Low-Dose 18F-Florbetaben Amyloid PET Imaging Using Deep Learning with Multi-Contrast MRI Inputs. Radiology.

[52] Wang Y., Yu B., Wang L., Zu C., Lalush D.S., Lin W., Wu X., Zhou J., Shen D., Zhou L. (2018). 3D Conditional Generative Adversarial Networks for High-Quality PET Image Estimation at Low Dose. NeuroImage.

[53] Zhuang Y., Mathai T.S., Mukherjee P., Summers R.M. (2024). Segmentation of Pelvic Structures in T2 MRI via MR-to-CT Synthesis. Comput. Med. Imaging Graph..

[54] Eshraghi Boroojeni P., Chen Y., Commean P.K., Eldeniz C., Skolnick G.B., Merrill C., Patel K.B., An H. (2022). Deep-Learning Synthesized Pseudo-CT for MR High-Resolution Pediatric Cranial Bone Imaging (MR-HiPCB). Magn. Reson. Med..

[55] Khan S.U., Ullah N., Ahmed I., Ahmad I., Mahsud M.I. (2019). MRI Imaging, Comparison of MRI with Other Modalities, Noise in MRI Images and Machine Learning Techniques for Noise Removal: A Review. Curr. Med. Imaging Rev..

[56] Domingues I., Pereira G., Martins P., Duarte H., Santos J., Abreu P.H. (2020). Using Deep Learning Techniques in Medical Imaging: A Systematic Review of Applications on CT and PET. Artif. Intell. Rev..

[57] Kinahan P.E., Hasegawa B.H., Beyer T. (2003). X-Ray-Based Attenuation Correction for Positron Emission Tomography/Computed Tomography Scanners. Semin. Nucl. Med..

[58] Israel O., Pellet O., Biassoni L., De Palma D., Estrada-Lobato E., Gnanasegaran G., Kuwert T., la Fougère C., Mariani G., Massalha S. (2019). Two Decades of SPECT/CT—The Coming of Age of a Technology: An Updated Review of Literature Evidence. Eur. J. Nucl. Med. Mol. Imaging.

[59] Balaji V., Song T.-A., Malekzadeh M., Heidari P., Dutta J. (2024). Artificial Intelligence for PET and SPECT Image Enhancement. J. Nucl. Med..

[60] Kao C.-H., Chen Y.-S., Chen L.-F., Chiu W.-C., de Bruijne M., Cattin P.C., Cotin S., Padoy N., Speidel S., Zheng Y., Essert C. (2021). Demystifying T1-MRI to FDG-18-PET Image Translation via Representational Similarity. Proceedings of the Medical Image Computing and Computer Assisted Intervention—MICCAI 2021.

[61] Hu Q., Li H., Zhang J., Wang L., Dou Q., Fletcher P.T., Speidel S., Li S. (2022). Domain-Adaptive 3D Medical Image Synthesis: An Efficient Unsupervised Approach. Proceedings of the Medical Image Computing and Computer Assisted Intervention—MICCAI 2022.

[62] Rajagopal A., Natsuaki Y., Wangerin K., Hamdi M., An H., Sunderland J.J., Laforest R., Kinahan P.E., Larson P.E.Z., Hope T.A. (2023). Synthetic PET via Domain Translation of 3-D MRI. IEEE Trans. Radiat. Plasma Med. Sci..

[63] Ahangari S., Beck Olin A., Kinggård Federspiel M., Jakoby B., Andersen T.L., Hansen A.E., Fischer B.M., Littrup Andersen F. (2022). A Deep Learning-Based Whole-Body Solution for PET/MRI Attenuation Correction. EJNMMI Phys..

[64] Chen K., Hosseini A.A., Weng Y., Dening T., Zuo G. (2023). Two-Stage Diffusion Model Deriving FDG-PET from T1 Weighted Magnetic Resonance Images for Diagnosis of Alzheimer’s Disease. Alzheimer’s Dement..

[65] Dong B., Zheng R., Sun X., Chen M., Li Q. (2024). Delineation of Primary Lung Cancer with Atelectasis Assisted by GANs-Based Synthetic PET Images from CT. Int. J. Radiat. Oncol. Biol. Phys..

[66] Lyu Q., Kim J.Y., Kim J., Whitlow C.T. (2024). Synthesizing Beta-Amyloid PET Images from T1-Weighted Structural MRI: A Preliminary Study 2024. arXiv.

[67] Zheng X., Worhunsky P., Liu Q., Zhou B., Chen X., Guo X., Xie H., Sun H., Zhang J., Toyonaga T. Generation of Synthetic Brain PET Images of Synaptic Density from MRI and FDG-PET Using a Multi-Stage U-Net. Proceedings of the 2024 IEEE Nuclear Science Symposium (NSS), Medical Imaging Conference (MIC) and Room Temperature Semiconductor Detector Conference (RTSD).

[68] Khojaste-Sarakhsi M., Haghighi S.S., Ghomi S.M.T.F., Marchiori E. (2024). A 3D Multi-Scale CycleGAN Framework for Generating Synthetic PETs from MRIs for Alzheimer’s Disease Diagnosis. Image Vis. Comput..

[69] Mansouri Z., Salimi Y., Akhavanallaf A., Shiri I., Teixeira E.P.A., Hou X., Beauregard J.-M., Rahmim A., Zaidi H. (2024). Deep Transformer-Based Personalized Dosimetry from SPECT/CT Images: A Hybrid Approach for [177Lu]Lu-DOTATATE Radiopharmaceutical Therapy. Eur. J. Nucl. Med. Mol. Imaging.

[70] Salehjahromi M., Karpinets T.V., Sujit S.J., Qayati M., Chen P., Aminu M., Saad M.B., Bandyopadhyay R., Hong L., Sheshadri A. (2024). Synthetic PET from CT Improves Diagnosis and Prognosis for Lung Cancer: Proof of Concept. Cell Rep. Med..

[71] Kobayashi T., Shigeki Y., Yamakawa Y., Tsutsumida Y., Mizuta T., Hanaoka K., Watanabe S., Morimoto-Ishikawa D., Yamada T., Kaida H. (2024). Generating PET Attenuation Maps via Sim2Real Deep Learning–Based Tissue Composition Estimation Combined with MLACF. J. Digit. Imaging. Inform. Med..

[72] Wang C., Piao S., Huang Z., Gao Q., Zhang J., Li Y., Shan H. (2024). Joint Learning Framework of Cross-Modal Synthesis and Diagnosis for Alzheimer’s Disease by Mining Underlying Shared Modality Information. Med. Image Anal..

[73] Lee J., Burkett B.J., Min H.-K., Senjem M.L., Dicks E., Corriveau-Lecavalier N., Mester C.T., Wiste H.J., Lundt E.S., Murray M.E. (2024). Synthesizing Images of Tau Pathology from Cross-Modal Neuroimaging Using Deep Learning. Brain.

[74] Enlow E., Abbaszadeh S. (2023). State-of-the-Art Challenges and Emerging Technologies in Radiation Detection for Nuclear Medicine Imaging: A Review. Front. Phys..

[75] Hosch R., Weber M., Sraieb M., Flaschel N., Haubold J., Kim M.-S., Umutlu L., Kleesiek J., Herrmann K., Nensa F. (2022). Artificial Intelligence Guided Enhancement of Digital PET: Scans as Fast as CT?. Eur. J. Nucl. Med. Mol. Imaging.

[76] Xie H., Gan W., Zhou B., Chen M.-K., Kulon M., Boustani A., Spencer B.A., Bayerlein R., Ji W., Chen X. (2024). Dose-Aware Diffusion Model for 3D Low-Dose PET: Multi-Institutional Validation with Reader Study and Real Low-Dose Data 2024. arXiv.

[77] Li Q., Zhu X., Zou S., Zhang N., Liu X., Yang Y., Zheng H., Liang D., Hu Z. (2022). Eliminating CT Radiation for Clinical PET Examination Using Deep Learning. Eur. J. Radiol..

[78] Ma K.C., Mena E., Lindenberg L., Lay N.S., Eclarinal P., Citrin D.E., Pinto P.A., Wood B.J., Dahut W.L., Gulley J.L. (2024). Deep Learning-Based Whole-Body PSMA PET/CT Attenuation Correction Utilizing Pix-2-Pix GAN. Oncotarget.

[79] Sanaat A., Shiri I., Arabi H., Mainta I., Nkoulou R., Zaidi H. Whole-Body PET Image Synthesis from Low-Dose Images Using Cycle-Consistent Generative Adversarial Networks. Proceedings of the 2020 IEEE Nuclear Science Symposium and Medical Imaging Conference (NSS/MIC).

[80] Zhou B., Miao T., Mirian N., Chen X., Xie H., Feng Z., Guo X., Li X., Zhou S.K., Duncan J.S. (2023). Federated Transfer Learning for Low-Dose PET Denoising: A Pilot Study with Simulated Heterogeneous Data. IEEE Trans. Radiat. Plasma Med. Sci..

[81] Fard A.S., Reutens D.C., Ramsay S.C., Goodman S.J., Ghosh S., Vegh V. (2024). Image Synthesis of Interictal SPECT from MRI and PET Using Machine Learning. Front. Neurol..

[82] Raymond C., Zhang D., Liu L., Moyaert P., Burneo J., Dada M., Hicks J., Finger E., Soddu A., Andrade A. (2024). Self-Similarity Awareness in PET Image Denoising: A Quantitative Evaluation of SMART-PET Framework for [18F]-FDG-PET Image Denoising. J. Nucl. Med..

[83] Shi Y., Xia W., Niu C., Wiedeman C., Wang G. (2023). Enabling Competitive Performance of Medical Imaging with Diffusion Model-Generated Images without Privacy Leakage 2024. arXiv.

[84] Pan S., Abouei E., Peng J., Qian J., Wynne J.F., Wang T., Chang C.-W., Roper J., Nye J.A., Mao H. (2024). Full-Dose Whole-Body PET Synthesis from Low-Dose PET Using High-Efficiency Denoising Diffusion Probabilistic Model: PET Consistency Model 2024. Med. Phys..

[85] Guan Y., Shen B., Jiang S., Shi X., Zhang X., Li B., Liu Q. (2024). Synthetic CT Generation via Variant Invertible Network for Brain PET Attenuation Correction. IEEE Trans. Radiat. Plasma Med. Sci..

[86] Shi L., Zhang J., Toyonaga T., Shao D., Onofrey J.A., Lu Y. (2023). Deep Learning-Based Attenuation Map Generation with Simultaneously Reconstructed PET Activity and Attenuation and Low-Dose Application. Phys. Med. Biol..

[87] Li W., Huang Z., Chen Z., Jiang Y., Zhou C., Zhang X., Fan W., Zhao Y., Zhang L., Wan L. (2024). Learning CT-Free Attenuation-Corrected Total-Body PET Images through Deep Learning. Eur. Radiol..

[88] Li X., Johnson J.M., Strigel R.M., Bancroft L.C.H., Hurley S.A., Estakhraji S.I.Z., Kumar M., Fowler A.M., McMillan A.B. (2024). Attenuation Correction and Truncation Completion for Breast PET/MR Imaging Using Deep Learning. Phys. Med. Biol..

[89] Wyatt J.J., Kaushik S., Cozzini C., Pearson R.A., Petrides G., Wiesinger F., McCallum H.M., Maxwell R.J. (2024). Evaluating a Radiotherapy Deep Learning Synthetic CT Algorithm for PET-MR Attenuation Correction in the Pelvis. EJNMMI Phys..

[90] Partin L., Spottiswoode B., Hayden C., Armstrong I., Fahmi R. (2024). Deep Learning-Based CT-Less Attenuation Correction of Brain FDG PET. J. Nucl. Med..

[91] Raj J., Millardet M., Krishnamoorthy S., Karp J.S., Surti S., Matej S. (2024). Recovery of the Spatially-Variant Deformations in Dual-Panel PET Reconstructions Using Deep-Learning. Phys. Med. Biol..

[92] Nagendran M., Chen Y., Lovejoy C.A., Gordon A.C., Komorowski M., Harvey H., Topol E.J., Ioannidis J.P.A., Collins G.S., Maruthappu M. (2020). Artificial Intelligence versus Clinicians: Systematic Review of Design, Reporting Standards, and Claims of Deep Learning Studies. BMJ.

[93] Hirata K., Matsui Y., Yamada A., Fujioka T., Yanagawa M., Nakaura T., Ito R., Ueda D., Fujita S., Tatsugami F. (2024). Generative AI and Large Language Models in Nuclear Medicine: Current Status and Future Prospects. Ann. Nucl. Med..

[94] Koitka S., Baldini G., Kroll L., van Landeghem N., Pollok O.B., Haubold J., Pelka O., Kim M., Kleesiek J., Nensa F. (2024). SAROS: A Dataset for Whole-Body Region and Organ Segmentation in CT Imaging. Sci. Data.

[95] Jung M., Raghu V.K., Reisert M., Rieder H., Rospleszcz S., Pischon T., Niendorf T., Kauczor H.-U., Völzke H., Bülow R. (2024). Deep Learning-Based Body Composition Analysis from Whole-Body Magnetic Resonance Imaging to Predict All-Cause Mortality in a Large Western Population. eBioMedicine.

[96] Xie S., Wu Z., Qi Y., Wu B., Zhu X. (2021). The Metastasizing Mechanisms of Lung Cancer: Recent Advances and Therapeutic Challenges. Biomed. Pharmacother..

